# Adherence to dietary guide for elderly adults and health risks of older adults in ethnic minority areas in China: a cross-sectional study

**DOI:** 10.1186/s12889-022-12668-1

**Published:** 2022-02-21

**Authors:** Tingyu Mai, Chunbao Mo, Jiansheng Cai, Haoyu He, Huaxiang Lu, Xu Tang, Quanhui Chen, Xia Xu, Chuntao Nong, Shuzhen Liu, Dechan Tan, Shengle Li, Qiumei Liu, Min Xu, You Li, Chunhua Bei, Zhiyong Zhang

**Affiliations:** 1 Department of Environmental Health and Occupational Medicine, School of Public Health, Guilin Medical University, Huan Cheng North 2nd Road 109, Guilin, 541004 Guangxi China; 2 School of Medicine, Southern University of Science and Technology, Shenzhen, 518055 Guangdong China; 3 Department of Environmental and Occupational Health, School of Public Health, Guangxi Medical University, Nanning, 530021 Guangxi China; 4 Department of Quality Management, The Affiliated Hospital of Stomatology, Guangxi Medical University, Nanning, 530021 Guangxi China; 5 Department of Guangxi Science and Technology Major Project, Guangxi Zhuang Autonomous Region Center for Diseases Control and Prevention, Nanning, 530028 Guangxi China; 6 Department of Hospital Infection-Control, Liuzhou Workers’ Hospital, Liuzhou, 545005 Guangxi China; 7 Nanning Municipal Center for Disease Control and Prevention, Nanning, 530023 Guangxi China; 8 Department of Epidemiology and Health Statistics, School of Public Health, Guilin Medical University, Huan Cheng North 2nd Road 109, Guilin, 541004 Guangxi China

**Keywords:** Older adults, Dietary guide for elderly adults, Adherence, TOPSIS, Ethnic minorities, Dietary pattern

## Abstract

**Background:**

The impact of dietary guidelines on health in ethnic minority regions needs to be further explored because of multiple sociocultural factors. Therefore, this study was conducted to analyze the association between adherence to dietary guidelines and health risks in an elderly population in an ethnic minority region.

**Methods:**

A cross-sectional survey was conducted among 836 older adults in ethnic minority areas. They were asked to describe their daily dietary intake levels through a semi-quantitative food frequency questionnaire. The closeness coefficient for each study subject was calculated by using the technique for order preference by similarity to an ideal solution (TOPSIS), which measures the adherence to *Dietary Guide for Elderly Adults* (DGEA). Regression models were used to analyze the association between adherence and health risks.

**Results:**

The daily food of the elderly in this area comprised cereals and vegetables. They had low intake of milk, dairy products, and water and high intake of salt. The closeness coefficient for the total population was 0.51, and the adherence of this population to dietary guidelines for the elderly was low. In both the crude model and the models adjusted for covariates, the closeness coefficient was not significantly associated with clinical indicators and health outcomes (*p* > 0.05).

**Conclusions:**

No association was found between adherence to large sample-based dietary guidelines and clinical indicators or health outcomes in ethnic minority populations. The applicability of dietary guidelines to ethnic minority areas and whether they yield the expected health benefits require further study.

**Supplementary Information:**

The online version contains supplementary material available at 10.1186/s12889-022-12668-1.

## Background

A balanced diet is the foundation of good health. The impact of diet on health was highlighted in 1998 when the Preparation and Use of Food-Based Dietary Guidelines were developed and promoted by WHO and FAO [1]. Since then, more than 100 countries around the world have formulated food source dietary guidelines that fit their own dietary culture. Countries have issued food guidelines in the form of food pyramids and food plates to guide consumers on how they can improve their daily eating habits. China’s dietary guidelines, first issued in 1989 and revised in 1997, 2007, and 2015 [2], have played an important role in guiding the development of a balanced diet and in preventing diseases.

A healthy diet reduces the risk of morbidity and mortality from chronic diseases and metabolic disorders [3]. Traditional studies have focused on the health effects of nutrients, components, or dietary patterns [3, 4], but current studies are beginning to focus on the health effects of adherence to dietary guidelines. Terumi Nishimura et al. observed that high adherence to the Japanese Food Guide Spinning Top was associated with lower waist circumference and LDL-C, but not with other metabolic indicators in a young Japanese female population [5]. According to a recent meta-analysis, adherence to the Alternative Healthy Eating Index (AHEI) and Dietary Approaches to Stop Hypertension (DASH) was associated with a 20% reduction in the risk of developing type 2 diabetes (T2D) [6], whereas adherence to the Mediterranean diet was associated with an 8% reduction in the risk of prediabetes (PreT2D) and a 50% reduction in the risk of T2D in a high-risk population [7, 8]. The above study used dietary guidance as a criterion to analyze the association between adherence to dietary guidance and population disease risk. These studies had two main objectives, as follows. First, they aimed to determine whether the dietary guidelines fit the purpose and resulted in the intended consequences. Second, they aimed to generate a reflection on the dietary guidelines and to further develop a more applicable one that is in line with local population’s characteristics. National Health Commission of the People’s Republic of China issued the *Dietary Guide for Elderly Adults* (DGEA) in the form of a health industry standard on 1 August 2017. It gives recommendations on daily food choices and quantities for convenient adoption and implementation by older adults in China. The current Chinese population is aging, and no study has reported the association between adherence to this guidance and health in the elderly population. In China, older adults in ethnic minority regions often have distinctive diets and living customs. The current Chinese population is aging, and no study has reported the association between adherence to this guidance and health risks in the elderly population. In China, older adults in ethnic minority regions often have distinctive diets and living customs. The ethnic minorities in the Gongcheng area of Guangxi are mainly Yao and Zhuang. Most Yao people live in deep mountains and are relatively isolated, which makes them different from the general population in terms of social culture and living habits [9]. Most of them intermarry within their ethnic groups and constitute an ideal group for genetic research. Thus, although guidelines have been developed to promote healthy eating among the older population, the health benefits of following the guidelines’ dietary habits remain largely unknown. Proven dietary guidelines can improve the health of the population. In the context of an aging population, public health resources can be used more efficiently, and life expectancy can improve. For policymakers, research based on these areas can make government efforts more efficient. In this cross-sectional study, we tested our basic hypothesis, that is, on the basis of the aforementioned findings, the adherence to the DGEA among the elderly population in ethnic minority areas is related to their clinical indicators or health outcomes. We also hope to evaluate whether DGEA applies to ethnic minority areas and produces the expected health benefits.

## Methods

### Study design and participants

**Fig. 1:**
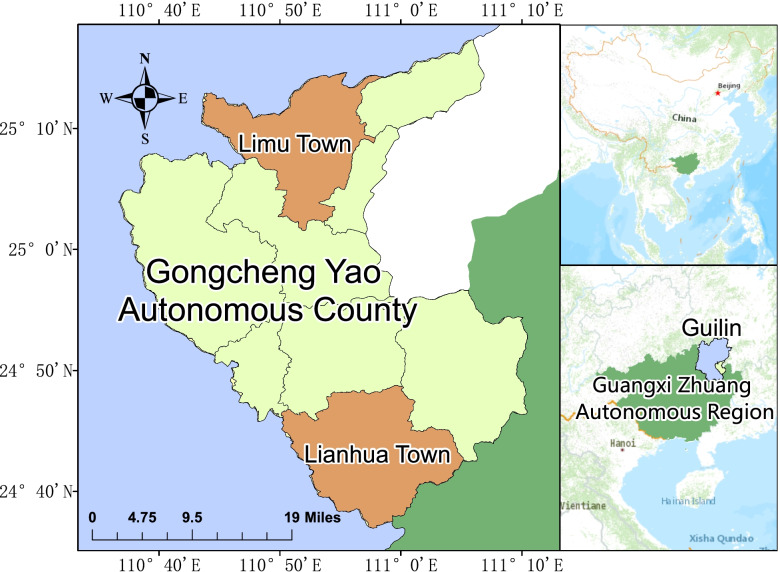
Cross-sectional study of the area and related geographical information and features. Footnotes: Guangxi Zhuang Autonomous Region is located in the south of China and covers an area of 237,600 km^2^. Guilin is a subordinate prefecture-level city in northeastern Guangxi, and there are several autonomous counties of ethnic minorities in Guilin. Gongcheng is a Yao autonomous county, located in the southeast of Guilin. Limu Town and Lianhua Town are the two larger townships in Gongcheng County

**Fig. 2:**
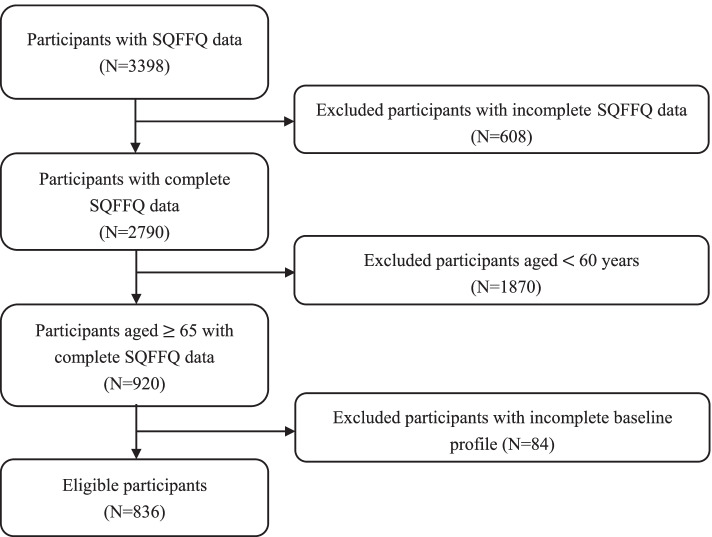
Flow chart of sample selection criteria: cross-sectional study. Footnotes: SQFFQ: semi-quantitative food frequency questionnaire

Guangxi is an important minority settlement in China. Gongcheng is a Yao autonomous county with an area of 2139 km^2^. It is located in the southeast of Guilin, Guangxi, and includes nine townships, including Lianhua and Limu (Fig. 1), with a resident population of 360,000, and Yao, Han, and Zhuang. From December 2018 to December 2019, we conducted a cross-sectional survey in the towns of Lianhua and Limu, where 3398 residents were routinely screened for health. A dietary assessment survey was conducted several months later. After excluding questionnaires with missing or abnormal information or with obvious errors, a total of 2790 participants were included for the meal frequency questionnaire. Among them were 920 elderly participants aged 65 years old and older. After excluding cases with missing medical examination data, a total of 836 individuals were included in this study. The above process is shown in Fig. 2. The cross-sectional study was approved by the Ethics Committee of Guilin Medical College, and all participants signed an informed consent form.

### Data collection

Volunteers made up of medical students were recruited and trained for one week to master the skills needed for this investigation. They were responsible for asking participants and filling out questionnaires for collecting information involving demographics, disease history, and nutritional dietary status. Participants were also required to undergo a routine physical examination, dual-energy x-ray absorptiometry (DXA) and abdominal ultrasound examination performed by a professional physician to collect anthropometric indicators, such as height, weight, blood pressure, bone mineral density (BMD), and the health of abdominal organs. Venous blood samples were collected and sent to a local hospital for biochemical testing. Routine physical examination included the determination of fasting plasma glucose (FPG), glycated hemoglobin (HbA1C), triglyceride (TG), total cholesterol (TC), high-density lipoprotein cholesterol (HDL-C), low-density lipoprotein cholesterol (LDL-C), and uric acid (UA). Parallel samples were set for all routine physical examination indexes at testing time.

### Definition

The BMD is divided into three status: normal, osteopenia, and osteoporosis, which are diagnosed by the interval of T-score using DXA. (1) Normal BMD is defined as a T-score at or above − 1.0 standard deviation (SD); (2) osteopenia is defined as a T-score between − 2.5 SD and − 1.0 SD, exclusively; and (3) osteoporosis is defined as a T-score at or below − 2.5 SD [10]. Fatty liver disease (FLD) is diagnosed by the presence of at least two of three abnormal findings on abdominal ultrasonography: liver anterior echogenicity (“bright liver”), far-field echo attenuation, and unclear intrahepatic duct structure [11]. However, hypertension, diabetes, cardiovascular and cerebrovascular disease, and cancer are defined as self-reported physician diagnosis (Supplemental Material).

### Dietary assessment

A semi-quantitative food frequency questionnaire (SQFFQ) was used to assess dietary intake. Volunteers were responsible for asking the participants about their dietary intake and filling out the SQFFQ to collect the frequency and single intake of each type of food in the past month. The SQFFQ consisted of 109 food items and included the following eight frequency categories: (1) rarely eat or drink; (2) less than once a week; (3) once a week; (4) 2–3 times a week; (5) 4–6 times a week; (6) once a day; (7) twice a day; and (8) thrice a day. The accuracy of the SQFFQ was ensured by using standard food charts and physical objects that allow for accurate weighing (to the nearest 0.1 g) as a reference. Finally, the total food intake over a period of time could be calculated from the frequency of food intake and the amount of individual servings, which further led to the daily grams of food intake. The 109 foods were then divided into 12 food groups according to the DGEA.

### Data normalization and TOPSIS

Before TOPSIS, other types of data (e.g., negative and interval data) needed to be normalized to make them positive, i.e., the larger the value is, the better it is. The interval data indicated that the closer the data is to a certain interval or even in a certain interval, the better the result. Some good examples include human body suitable temperature, acidity, and others. According to the above definition, the daily dietary intake belonged to the interval type data. We determined the recommended daily intake of various foods by referring to the DGEA. For example, the recommended daily intake of vegetables was 300 g to 400 g (Table 1). The recommended intakes for each food group were normalized to meet the TOPSIS requirements with the following formula.

$$M=\max \left\{\ a-\mathit{\min}\left\{{x}_i\right\},\mathit{\max}\left\{{x}_i\right\}-b\right\},{x}_i^{\prime }=\left\{\begin{array}{c}1-\frac{a-{x}_i}{M},{x}_i<a\ \\ {}\ 1,a\le {x}_i\le b\ \\ {}1-\frac{x_i-b}{M},{x}_i>b\ \end{array}\right.$$

**Table 1:** Daily Recommended Nutrient Intake of 12 Food Groups Recommended in the DGEA

Food category		Daily recommended nutrient intake
Cereals and potatoes	Male	250 g ~ 300 g
	Female	200 g ~ 250 g
Vegetables		300 g ~ 400 g
Fruit		100 g ~ 200 g
Meat		40 g ~ 50 g
Aquatic product and poultry		50 g ~ 100 g
Soybean and nuts		30 g ~ 50 g
Eggs		25 g ~ 50 g
Milk and milk products		250 g ~ 300 g
Oil		20 g ~ 25 g
Salt		< 5 g
Water		1.5 L ~ 1.7 L
Alcohol	Male	< 25 g
	Female	< 15 g

*DGEA* Dietary Guide for Elderly Adults

In the formula, a and b denote the recommended intake interval range [a, b] for a nutrient, *x*_*i*_ denotes the value that needs to be normalized, and ${x}_i^{\prime }$ denotes the normalized *x*_*i*_. After using the formula, y ranged from 0 to 1. Therefore, when the value of ${x}_i^{\prime }$ is larger, the *x*_*i*_ is closer to the reference standard [a, b], which means that the daily intake of such food is more in line with the recommended daily intake in the dietary guidelines. When ${x}_i^{\prime }$ =1, the daily intake of such food is in full compliance.

The TOPSIS method was used to comprehensively evaluate the intake of 12 food groups for each study subject. The basic principle of TOPSIS is as follows. The best and the worst of the limited solutions constitute a space. A solution to be evaluated can be regarded as a point in the space. By obtaining the distance between this point and the optimal and the worst solutions, the closeness coefficient (*C*_*i*_) can be obtained to evaluate the merits and demerits of the solution. TOPSIS has been used in many fields, such as healthcare [12, 13], environment [14], and security [15]. The TOPSIS method avoids the subjective bias associated with scoring methods by providing a comprehensive evaluation of normalized data, and the closeness coefficients in TOPSIS results represent a more objective view of dietary adherence. The closeness coefficient can reflect its distance from the (positive) ideal solution and the negative ideal solution, which is in line with people’s conventional logical thinking. In this study, we hope to evaluate the applicability of this method in dietary research. The calculation steps are as follows:

1. Construct the judgment matrix: *A* = (*x*′_*ij*_)*m* × *n (i = 1, 2, …, m; j = 1, 2, …, n)*
2. According to the judgment matrix A, determine the optimal solution Q+ and the worst solution Q-: ${Q}_{+}=\left({r}_1^{+},{r}_2^{+},\dots, {r}_n^{+}\right),{Q}_{-}=\left({r}_1^{-},{r}_2^{-},\dots, {r}_n^{-}\right)$  
3. Calculate the distance ${S}_i^{+}$ and ${S}_i^{-}$ of each scheme from Q_ + and Q_-:

$${S}_i^{+}=\sqrt{\sum_{j=1}^n{\left({r}_{ij}-{r}_j^{+}\right)}^2},{S}_i^{-}=\sqrt{\sum_{j=1}^n{\left({r}_{ij}-{r}_j^{-}\right)}^2}$$

4. Calculate the closeness coefficient *C*_*i*_ of each solution to the optimal solution:

$${C}_i={S}_i^{+}/\left({S}_i^{+}+{S}_i^{-}\right)$$

In this formula, *C*_*i*_ ∈ [0, 1], a greater closeness coefficient indicated that the intake of the 12 diets of the participant was more in line with the DGEA, which meant higher adherence.

### Statistical analysis

Continuous variables were expressed using the mean ± standard deviation $\left(\overline x\;\pm\;\text{s}\right)$, whereas categorical variables were described using the number of cases and composition ratio. The association between closeness coefficient and each health indicator was assessed by developing multiple linear regression or logistic regression models. Age, gender, ethnicity, marital status, education level, occupation, household income, and smoking were selected as covariates based on previous studies and creation of a directed acyclic graph (Supplemental Material). We used three models, as follows. The first model was a crude one without covariates, and the second included gender and age as covariates in the adjustment. The third adjustment model adds the other remaining covariates mentioned above. A bootstrap procedure with 1000 substitutions was performed for more robust estimations. *P* < 0.05 was considered statistically significant. We performed simple calculations and normalized the data with Excel 2020, then performed TOPSIS analysis using R software 4.0.2. Finally, we constructed regression models using SPSS 26.0 (IBM, Chicago, IL, USA).

## Results

### Characteristics of the study population

Table 2 shows the results of the demographic characteristics of the study population. The mean age of the participants was 71.36 years old; 56.0% of the participants were male. Yao and Han were the predominant ethnic groups (60.3 and 34.2%, respectively). The vast majority of participants were married (96.5%); 95.5% had an educational level that was less than high school. Most participants were engaged in agricultural production activities (91.4%). For household income, most participants were earning ≥$5000/year. Most participants did not smoke or drink alcohol (79.9 and 62.9%, respectively).

**Table 2:** The Demographic Characteristic for Older People Enrolled in the Cross-sectional Study (*n* = 836)

Characteristics		Mean ± SD or n (%)
Sample size		836
Age (years old)		71.36 ± 4.67
Gender	Male	468 (56.0)
	Female	368 (44.0)
Nation	Yao	504 (60.3)
	Han	286 (34.2)
	Zhuang	29 (3.5)
	Other	2 (0.2)
Married	Yes	807 (96.5)
	No	11 (1.3)
Education level	<high school	798 (95.5)
	≥high school	23 (2.8)
Occupation	Farmer	764 (91.4)
	Other	49 (5.9)
Household income (RMB/year)	<5000	280 (33.5)
	≥5000	542 (64.7)
Smoking	Yes	153 (18.3)
	No	668 (79.9)
Drinking	Yes	295 (35.3)
	No	526 (62.9)

Table 3 demonstrates the results of the health indicators in the study population, which were within the normal range except for the mean values of SBP (144.84 mmHg), TC (5.67 mmol/L), and LDL.C (3.61 mmol/L), which were higher than the normal values. In addition, except for the high percentage of osteoporosis (40.0%), the percentages of all other diseases were relatively low.

**Table 3:** The Clinical Indicators or Health Outcomes for Older People Enrolled in the Cross-sectional Study (*n* = 836)

Characteristics		Mean ± SD or n (%)
Sample size		836
BMI		21.75 ± 3.49
SBP		144.84 ± 26.10
DBP		84.26 ± 15.52
FPG		5.33 ± 1.56
HbA1C		6.02 ± 0.98
TC		5.67 ± 1.07
TG		1.28 ± 1.03
LDL.C		3.61 ± 0.97
HDL.C		1.84 ± 0.45
UA		319.60 ± 95.41
BMD	Normal	341 (40.8)
	Osteopenia	161 (19.3)
	Osteoporosis	334 (40.0)
FLD	Yes	95 (11.4)
	No	741 (88.6)
Hypertension	Yes	252 (30.1)
	No	569 (68.1)
Diabetes	Yes	39 (4.7)
	No	782 (93.5)
CCD	Yes	51 (6.1)
	No	770 (92.1)
Cancer	Yes	2 (0.2)
	No	819 (98.0)

Footnotes: *BMI* Body mass index, *SBP/DBP* Systolic/diastolic blood pressure (mmHg), *FPG* Fasting plasma glucose (mmol/L), *HbA1C* Glycosylated hemoglobin (%), *TC* Total cholesterol (mmol/L), *TG* Triglyceride (mmol/L), *LDL-C* Low-density lipoprotein cholesterol (mmol/L), *HDL-C* High-density lipoprotein cholesterol (mmol/L), *UA* Uric acid (μmol/L), *BMD* Bone mineral density, *FLD* Fatty liver disease, *CCD* Cardiovascular and cerebrovascular disease

### Population diet and TPOSIS results

Using semi-quantitative food frequency tables, we calculated and normalized the daily intake of each food group for each study subject. Based on the results, TOPSIS analysis was performed, and the closeness coefficient was derived. The results are shown in Table 4. The average daily intake values of cereals and vegetables in this population were 310.68 and 300.37 g, respectively. The average daily intake values of fish and poultry (23.27 g), milk and milk products (31.66 g), and water (187.79 ml) were significantly lower than the recommended intake values. In contrast, the average daily intake of salt was significantly higher than the recommended intake at 9.05 g.

**Table 4:** Average Daily Intake of the 12 Food Groups in the Subject Population (*N* = 836), and Average Closeness Coefficient

Characters	Daily Intake	Normalized Values	Closeness Coefficient
Cereals and potatoes	310.68 ± 209.30	0.91 ± 0.12	0.51 ± 0.10 (0.423, 0.933) ^a^
Vegetables	300.37 ± 303.49	0.94 ± 0.07	
Fruit	188.44 ± 220.11	0.95 ± 0.09	
Meat	50.09 ± 76.26	0.96 ± 0.07	
Aquatic product and poultry	23.27 ± 32.06	0.83 ± 0.09	
Soybean and nuts	21.45 ± 34.38	0.93 ± 0.08	
Eggs	30.53 ± 32.88	0.84 ± 0.13	
Milk and milk products	31.66 ± 69.40	0.13 ± 0.23	
Oil	31.31 ± 25.95	0.95 ± 0.08	
Salt	9.05 ± 7.88	0.95 ± 0.08	
Water	187.79 ± 408.38	0.74 ± 0.06	
Alcohol	13.45 ± 33.85	0.97 ± 0.10	

Footnotes: A greater closeness coefficient indicated that the intake of the 12 diets of the participant was more in line with the DGEA, which meant higher adherence.

^a^(min, max)

Table 4 shows that the mean normalized values for most food groups were greater than 0.9, but some values were lower, such as those for milk and milk products (0.13) and water intake (0.74). These findings indicated that the intake of the above two food groups in this population was far from the recommended standard, especially the former. We used TOPSIS to calculate the closeness coefficient of 12 food groups for each study participant as a composite evaluation index. The largest closeness coefficient was 0.933, whereas the smallest was 0.423, and the mean value was 0.51. These data indicated that the overall dietary intake habits of this population had a large gap with the recommended intake standard when analyzed by combining the 12 food groups, thereby indicating low adherence.

### Regression analysis results

Regression analysis was performed using the closeness coefficient as the independent variable, and clinical indicators or health outcomes as the dependent variables. The analysis results are presented in Table 5. No significant association was found between the closeness coefficient and clinical indicators or health outcomes in the three models (*p* > 0.05), and bootstrapped estimation also yielded the same results. Notably, in adjusted Model 2, coefficient β = 2.272 and *p* = 0.019, which may indicate that the association between closeness coefficient and FLD was significant; however, the span of the 95% confidence interval (95% CI = 1.442, 65.243) was extremely large to be stable. Further analysis via bootstrapped estimation showed that 95% CI (− 0.046, 4.062) included 1. Hence, we regarded that the association between closeness coefficient and FLD was not perceived as significant.

**Table 5:** Association Between Adherence to DGEA and Clinical Indicators or Health Outcomes in the Subject Population (*n* = 836)

	Crude model			Adjusted model 1					Adjusted model 2				
	Linearized Estimation			Linearized Estimation			Bootstrapped Estimation		Linearized Estimation			Bootstrapped Estimation	
	*β* ^a^	*95% CI*	*P*	*β* ^a^	*95% CI*	*P*	*95% CI*	*P*	*β* ^a^	*95% CI*	*P*	*95% CI*	*P*
HbA1C	0.181	−0.485 - 0.847	0.594	0.220	−0.447 - 0.887	0.518	− 0.451 - 1.067	0.653	0.244	− 0.499 - 0.986	0.519	− 0.500 - 1.292	0.629
TC	−0.269	− 0.997 - 0.459	0.468	− 0.244	− 0.959 - 0.471	0.503	−0.896 - 0.339	0.406	−0.269	−1.041 - 0.502	0.493	−0.980 - 0.424	0.456
TG	0.035	−0.668 - 0.737	0.923	0.068	−0.633 - 0.769	0.849	−0.695 - 0.998	0.856	−0.118	−0.832 - 0.596	0.746	−0.864 - 0.786	0.782
LDL.C	−0.190	−0.852 - 0.471	0.572	−0.172	−0.831 - 0.487	0.609	−0.827 - 0.510	0.585	−0.164	−0.870 - 0.542	0.648	−0.937 - 0.508	0.650
HDL.C	−0.076	−0.384 - 0.232	0.628	−0.077	−0.384 - 0.230	0.625	−0.345 - 0.176	0.564	−0.109	−0.441 - 0.223	0.521	−0.417 - 0.174	0.480
UA	−19.955	−84.901 - 44.991	0.547	−22.856	−83.476 - 37.763	0.459	−66.085 - 34.384	0.311	−24.409	−90.004 - 41.186	0.465	−71.573 - 29.854	0.336
FPG	0.224	−0.837 - 1.285	0.678	0.197	−0.866 - 1.261	0.716	−0.862 - 1.441	0.794	0.286	−0.861 - 1.433	0.625	−0.970 - 1.753	0.699
SBP	−0.033	−17.801 - 17.735	0.997	−2.071	− 19.788 - 15.646	0.819	−19.306 - 15.787	0.818	3.147	−15.886 - 22.180	0.746	−17.032 - 24.218	0.726
DBP	−3.482	−14.046 - 7.083	0.518	−3.655	−14.251 - 6.941	0.499	−12.126 - 4.788	0.397	−2.450	−13.934 - 9.034	0.675	−12.147 - 7.194	0.600
BMI	0.144	−2.235 - 2.524	0.905	0.384	−1.986 - 2.754	0.750	−2.341 - 3.411	0.775	−0.116	−2.555 - 2.323	0.926	−3.347 - 3.122	0.934
BMD	0.903	−0.375 - 2.181	0.166	0.796	−0.616 - 2.208	0.269	−0.532 - 2.190	0.677	0.766	−0.755 - 2.287	0.323	−0.662 - 2.217	0.714
FLD	1.589	0.795–30.177	0.087	1.805	0.962–38.471	0.055	−0.553 - 3.635	0.050	2.272	1.442–65.243	0.019	−0.046 - 4.062	0.019
Hypertension	−0.400	0.147–3.062	0.606	−0.540	0.126–2.689	0.489	−2.310 - 0.994	0.514	−0.401	0.132–3.393	0.628	−2.435 - 1.204	0.644
Diabetes	0.047	0.043–25.507	0.977	0.334	0.055–35.809	0.840	−6.202 - 2.879	0.856	0.543	0.062–47.955	0.749	−7.837 - 3.163	0.770
CCD	−0.260	0.041–14.446	0.862	− 0.307	0.039–13.964	0.838	−3.887 - 1.845	0.814	0.008	0.051–19.751	0.996	−3.393 - 2.280	0.997
Cancer ^b^													

Footnotes: *HbA1C* Glycosylated hemoglobin (%), *TC* Total cholesterol (mmol/L), *TG* Triglyceride (mmol/L), *HDL-C* High-density lipoprotein cholesterol (mmol/L), *LDL-C* Low-density lipoprotein cholesterol (mmol/L), *UA* Uric acid (μmol/L), *FPG* Fasting plasma glucose (mmol/L), *SBP/DBP* Systolic/diastolic blood pressure (mmHg), *BMI* Body mass index, *BMD* Bone mineral density, *FLD* Fatty liver disease, *CCD* Cardiovascular and cerebrovascular disease

*DGEA* Dietary Guide for Elderly Adults

^a^ Regression coefficients for the analysis with adherence as the independent variable and clinical indicators or health outcomes as the dependent variable

^b^ The number of cancer cases was too small to calculate

Crude model: no adjustment; Adjusted model 1: adjusted for age and gander; Adjusted model 2: adjusted for age, gander, nation, marital status, education level, occupation, household income and smoking

## Discussion

Cereals and vegetables were the main daily food items of the elderly in the region, with average daily intake values of 310.68 and 300.37 g, respectively. This finding was roughly in line with the intake recommended in the DGEA, as follows: cereal intake among males was 250–300 g/d, whereas that among females was 200–250 g/d; and vegetable intake was 300–400 g/d. In China, especially in the central and southern regions, cereals and vegetables have always been the main source of daily food, as determined by the suitability of crops, such as cereals and vegetables, for cultivation in these regions and the dietary habits of the population. This study was conducted in an area that was not suitable for livestock farming and where milk and its products were not traditionally consumed. Thus, the average daily intake of milk and its products by the elderly population in this area was only 31.66 g, which was far below the recommended intake of 250–300 g/d. Residents habitually drink homemade oil tea (a soup comprising tea, ginger, salt, oil, and water), which has good health benefits [16, 17]. Most residents drink oil tea in the morning, midday, and evening. To some extent, this habit affected the amount of water an individual actively drank daily; 1500–1700 ml/d of plain water was the recommended consumption. Notably, the average daily intake of salt (9.05 g/d)) was nearly twice the recommended standard (less than 5.0 g/d), indicating a high intake of salt by the population in the area. This situation was similar to that of other ethnic minority areas, and an epidemiological survey of Nandan County, an ethnic minority area in Guangxi, showed that the daily salt intake of the local Baiku Yao and Han populations was high (7.17 and 7.77 g/d, respectively) [18]. In addition, Emily’s survey of the Kunge Community in Yunnan’s ethnic minority areas also showed a high proportion of people with high-salt diet (53.9%) [19], This finding was attributed to the dietary habits of the local population.

Dietary guideline compliance affects food selection and quantity, according to particular rules of DGEA. The level of adherence reflects the degree of generalization of dietary guidelines. China is a multi-ethnic country, and some ethnic minorities have their own unique dietary culture and living habits. The dietary guidelines developed based on a large national sample lacked consideration of the differences among ethnic groups, which is the main reason for the low mean closeness coefficient of the total population. This finding indicated that the overall adherence to the dietary guidelines for the elderly is low. In addition, no significant association was found between adherence and changes in health risks. A 2008 meta-analysis showed that adherence to the Mediterranean diet is associated with a lower incidence of chronic diseases [20]. The association between adherence to the Mediterranean diet and a lower incidence of cancer came from a meta-analysis in 2020 [21]. A cohort study conducted in the United States since 1976 showed that greater adherence to the Mediterranean diet is associated with a lower risk of coronary heart disease and stroke in women [22]. Interestingly, the findings of studies on the association of adherence with disease and health status have not always been positive. Some studies have indicated that adherence to the Mediterranean diet is not associated with the prevalence of metabolic syndrome [23] and overall cancer risk [24]. This finding was attributed to the differences between the study groups. Geographical or ethnic differences indicated diverse dietary habits. If residents were guided by a specific dietary guideline (e.g., the Mediterranean diet), then the guideline may not have a full effect on health. The local government needs to further adjust the dietary guidelines according to the local dietary habits to make such guidelines more effective. Existing studies have highlighted the influences of cultural differences among different ethnic groups on dietary habits. Two qualitative studies conducted in the Netherlands analyzed active consumption and passive care diets with immigrants as the main ethnic minority [25, 26]. In 2016, a review of European immigrants reported that social and cultural environmental factors accounted for the highest proportion of factors influencing dietary behavior [27]. In addition, some studies have shown that cultural background determines food choices, certain beliefs about health behaviors, and strong eating habits. For example, one study conducted in the Netherlands indicated that minority perceptions of healthy foods were based on cultural background influences [25] and that traditions of hospitality influence dietary intake to some extent [26]. Cultural background also seems to determine the influence of social environment on dietary intake [28]. A study of the dietary habits of ethnic minorities helps elucidate how their societies and cultures function and the role of individuals and can explain the mechanisms by which behavioral norms, such as religion and customs, work in ethnic minority groups from another perspective [27]. Different from European studies, the Yao and Zhuang populations are not immigrants, and they do not feel alienated by differences in cultural background and dietary habits. Being located in an autonomous minority region, the unique culture has been well preserved, and many of the health practices unique to the region (Yao medicine) have been carried forward. The relationship between dietary compliance and health may be influenced by the long-term consumption of specialty foods (oil tea) and the maintenance of traditional lifestyles by the elderly population. The hilly terrain of the region has, to some extent, hindered its communication with the outside world while ensuring cultural stability and uniqueness. On the basis of the aforementioned discussion, the low dietary adherence of the elderly population in the region can be explained. For policy-makers, health promotion programs should be culturally sensitive, fit the diet habits and social environment of specific ethnic groups, and increase their social support and acceptance [29].

Challenges exist in the quantitative evaluation of adherence to food-based dietary guidelines. An early scale used to evaluate adherence to the traditional Mediterranean diet was proposed by Trichopoulou et al. The Mediterranean Diet Score was used to study the association between adherence to the traditional Mediterranean diet and a reduction in disease or mortality [22, 30]. The modified Alternate Mediterranean Diet (aMed) Score was extended to non-Mediterranean countries and regions [31]. Similarly, the Dutch Healthy Diet index (DHD-index) exists for the Dutch Dietary Guidelines. However, these methods have drawbacks, such as limited applicability, difficulty in replication, and unstable results. In this study, we used TOPSIS to evaluate adherence. The basic idea of this method is to normalize the data and to synthesize the evaluation by combining multiple food groups (indicators) into one indicator of collective closeness, which is used to directly measure the level of adherence. TOPSIS is a simple, logical, objective, easy-to-generalize, and highly applicable technique. In addition, in a practical sense, the method considers the potential interaction or synergistic effects of various foods. Combining the above discussion and results, we believe that no association between adherence to the DGEA and clinical indicators or health outcomes in the elderly population in Gongcheng County, Guangxi.

Two points should be noted in this study, as follows. (1) We only selected “food selection for the elderly” in the DGEA. However, two other components should be included, namely, the principles of DGEA, and the energy and major nutrient reference intakes for the elderly. For the general population rather than nutrition professionals, the phrase “food choices for the elderly” provides a more realistic guidance, is more operable, and is easier to promote. It plays an irreplaceable role in guiding the food choices and consumption quantities of the elderly. Therefore, the adherence in this study focused on the food choices of older adults. (2) The guidelines did not give a clear recommended lower limit for salt and alcohol intake. Thus, the default range of salt intake in this study was 0–5.0 g/d, and the range of alcohol intake was 0–25 g/d for men and 0–15 g/d for women. However, in practice, salt intake is not as low as possible. Thus, further studies are needed to clarify a reasonable recommended intake range [32].

In addition, this study had the following limitations. (1) The sample size of this study was small, which may have reduced the strength and significance of the association between adherence and health indicators. (2) This study used a retrospective dietary frequency survey, which made it difficult to avoid the shortcoming of accuracy due to forgetfulness. (3) The indicators reflecting clinical indicators or health outcomes in this study only represented a short time status and were not comprehensive. (4) The cross-sectional findings had limited probative power, and the results might be questioned. (5) The TOPSIS method did not consider the weighing of food types and other covariates that might be related to clinical indicators or health outcomes, such as exercise, and the results might be underestimated. Therefore, a better and more rational experimental design should be used when conducting research studies on the association between dietary adherence and health factors, especially in ethnic minority areas, such as Guangxi, China.

## Conclusion

The current cross-sectional study showed no association between adherence to the Dietary Guide for Elderly Adults and clinical indicators or health outcomes in the elderly population in Gongcheng County, Guangxi. Further expansion of the sample size is needed to accumulate scientific evidence on such an association.

## Supplementary Information

**Additional file 1.** Supplemental material for this article is available online.

## Data Availability

The datasets used and/or analyzed during the current study are available from the corresponding author on reasonable request.

## Abbreviations

AHEI: Alternative healthy eating index
DASH: Dietary approaches to stop hypertension
T2D: Type 2 diabetes
DGEA: Dietary guide for elderly adults
TOPSIS: Technique for order preference by similarity to an ideal solution
FPG: Fasting plasma glucose
HbA1C: Glycated hemoglobin
TG: Triglyceride
TC: Total cholesterol
HDL-C: High-density lipoprotein cholesterol
LDL-C: Low-density lipoprotein cholesterol
SQFFQ: Semi-quantitative food frequency questionnaire
UA: Uric acid (μmol/L)
FPG: Fasting plasma glucose (mmol/L);
SBP/DBP: Systolic/diastolic blood pressure (mmHg)
BMI: Body mass index
BMD: Bone mineral density
FLD: Fatty liver disease
CCD: Cardiovascular and cerebrovascular disease
