# Supplementary material for: Adherence to dietary guide for elderly adults and health risks of older adults in ethnic minority areas in China: a cross-sectional study
Source: BMC Public Health. 2022 Feb 21;22:372. doi: 10.1186/s12889-022-12668-1 (PMC8862314; doi:10.1186/s12889-022-12668-1)

**Diagnosis**

Hypertension is defined as self-reported physician diagnosis of hypertension referencing the criteria: 1) a clinic systolic blood pressure (BP) ≥ 140mmHg and/or diastolic BP ≥ 90 mmHg without the use of anti-hypertensive medications at three visits on different days. 2) or subjects with a BP < 140/90 mmHg but having hypertensive history and currently are taking anti-hypertensive medication^1^.

Diabetes is defined as self-reported physician diagnosis of diabetes referencing the criteria: anyone with HbA1c ≥ 6.5%, fasting plasma glucose (FPG) ≥ 7.0 mmol/l, OGTT 2 hours post-load plasma glucose (P2hPG) ≥ 11.1 mmol/l or a previous diagnosis of diabetes was defined as diabetic^2^.

Cardiovascular and cerebrovascular disease (CCD) is defined as self-reported physician diagnosis with at least one cardiovascular or cerebrovascular diseases, such as atherosclerosis, coronary heart disease, and cerebral ischemic stroke.

Cancer is defined as self-reported physician diagnosis with at least one cancers, such as lung, nasopharynx and other cancer.

Reference

1. Revision JCFG. 2018 Chinese Guidelines for Prevention and Treatment of Hypertension-A report of the Revision Committee of Chinese Guidelines for Prevention and Treatment of Hypertension. *Journal of geriatric cardiology : JGC* 2019; 16. DOI: 10.11909/j.issn.1671-5411.2019.03.014.

2. Association AD. Diagnosis and classification of diabetes mellitus. *Diabetes Care* 2006; 29 Suppl 1.

Figure S1. The directed acyclic graph for all variables (abbreviated version).


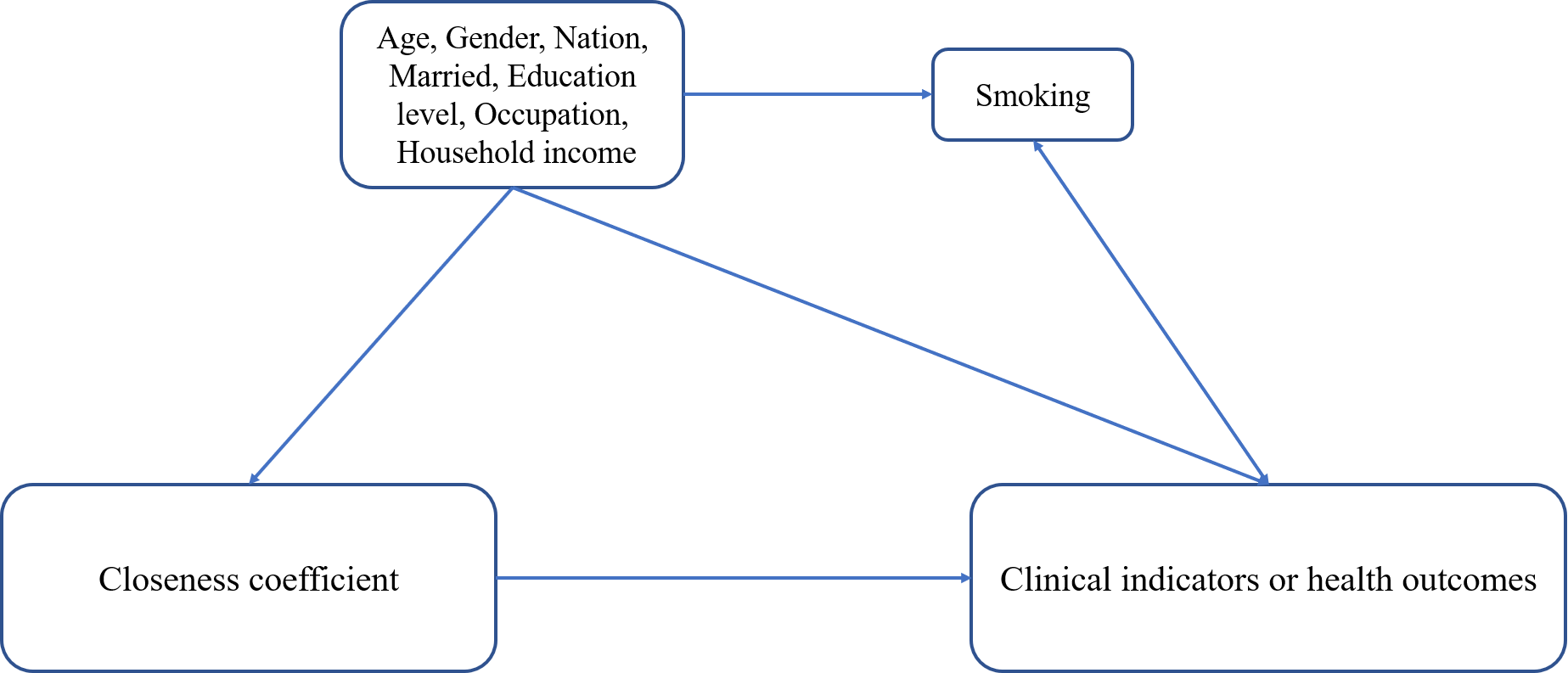

Supplement: Supplementary file 1 — Additional file 1. Supplemental material for this article is available online. [file 12889_2022_12668_MOESM1_ESM.docx]
